# Systematic Review of Clinical Practice Guidelines Related to Multiple Sclerosis

**DOI:** 10.1371/journal.pone.0106762

**Published:** 2014-10-10

**Authors:** Jia Guo, Chuang Cheng, Weiping Yan, Guanghui Xu, Jinzhou Feng, Tianzhu Wang, Cindy Si Chen, Xinyue Qin

**Affiliations:** 1 Department of Neurology, The First Affiliated Hospital of Chongqing Medical University, Chongqing, PR China; 2 Department of Neurology, The Third People's Hospital of Chengdu, Chengdu, PR China; 3 Department of Medicine, Drexel University College of Medicine, Philadelphia, Pennsylvania, United States of America; Hospital Nacional de Parapléjicos - SESCAM, Spain

## Abstract

**Background:**

High quality clinical practice guidelines (CPGs) can provide clinicians with explicit recommendations on how to manage health conditions and bridge the gap between research and clinical practice. Unfortunately, the quality of CPGs for multiple sclerosis (MS) has not been evaluated.

**Objective:**

To evaluate the methodological quality of CPGs on MS using the AGREE II instrument.

**Methods:**

According to the inclusion and exclusion criteria, we searched four databases and two websites related to CPGs, including the Cochrane library, PubMed, EMBASE, DynaMed, the National Guideline Clearinghouse (NGC), and Chinese Biomedical Literature database (CBM). The searches were performed on September 20th 2013. All CPGs on MS were evaluated by the AGREE II instrument. The software used for analysis was SPSS 17.0.

**Results:**

A total of 27 CPGs on MS met inclusion criteria. The overall agreement among reviews was good or substantial (ICC was above 0.70). The mean scores for each of all six domains were presented as follows: scope and purpose (mean ± SD: 59.05±16.13), stakeholder involvement (mean ± SD: 29.53±17.67), rigor of development (mean ± SD: 31.52±21.50), clarity of presentation (mean ± SD: 60.39±13.73), applicability (mean ± SD: 27.08±17.66), editorial independence (mean ± SD: 28.70±22.03).

**Conclusions:**

The methodological quality of CPGs for MS was acceptable for scope, purpose and clarity of presentation. The developers of CPGs need to pay more attention to editorial independence, applicability, rigor of development and stakeholder involvement during the development process. The AGREE II instrument should be adopted by guideline developers.

## Introduction

Multiple sclerosis (MS) is a chronic disease that attacks the central nervous system, i.e. the brain, spinal cord and optic nerves. It is characterized by the destruction of the myelin sheath that surrounds neurons, resulting in the formation of plaques. The cause of MS is unknown. One of the widely supported hypotheses is that MS occurs in patients with genetic susceptibility and is triggered by certain environmental factors. Recent data shows that in the USA over 350,000 people have MS, and a report from Cleveland Clinic indicates that MS-related health care costs are thought to be over $10 billion per year in the United States alone. Symptoms usually appear initially between 15 and 45 years of age. Women are presently twice as likely to get MS as men [1].

In the past, the decisions for diagnosis and treatment in any disease, including MS, were primarily based on a physician's experience rather than on evidence. The resultant variability in clinical practice was recognized by medical organizations and consensus meetings were conducted to develop recommendations [2].

The intention of clinical practice guidelines (CPGs) is to provide clinicians with explicit recommendations on how to manage health conditions and bridge the gap between research and clinical practice [3]. Unfortunately, it is difficult to gauge how a guideline is applied and performs in clinical practice [4]. Of the CPGs used in 235 studies assessing the effectiveness and efficiency of dissemination and implementation strategies, only 3% of the guidelines used were based on good evidence [5]. A “good” guideline should be scientifically valid, usable, reliable, and should improve the outcome of patients [4]. Standards are needed to promote the rigorous development of such guidelines, which should also be internationally recognized and feasible [6].

The Appraisal of Guidelines, Research, and Evaluation (AGREE) instrument evaluates the process of CPG development and reporting quality based on theoretical assumptions [7]. The AGREE instrument was initially developed in 2003, and updated to AGREEII in 2010, consisting of 23 key items organized into 6 domains [8]. The last update of AGREE II was September 2013.

To our knowledge, there has been no critical evaluation performed regarding guidelines or consensus on management of MS. We have, therefore, evaluated the methodological quality with the AGREE II instrument. In addition, we compared the quality of CPGs according to different stratified factors including year of publication, country/region, level of development, number of authors, topics covered, type of CPGs, etc.

## Methods

### Eligibility criteria

We included guidelines/consensuses that provided recommendations on diagnosis, treatment, and management of MS. For inclusion in our study, the CPGs were required to (1) be published in English and Chinese, and (2) to explicitly identify itself as a “guideline” or “consensus”. When more than one set of guidelines were produced by the same working groups or covered the same topics, only the most recently issued was considered; and (3) the cutoff time for inclusion of CPGs was September 2013. We excluded guidelines that (1) were Chinese versions of foreign CPGs and consensuses and adapted version of CPGs from other countries; (2) were duplications; and (3) were explanations or evaluations of CPGs.

### Information sources

Medical Subject Headings and text words related to multiple sclerosis and guidelines were used to search in four databases and two websites related to CPGs, which included PubMed (1966–2013.9), EMBASE.com (1974–2013.9), Cochrane Library (−2013.9), and Chinese Biomedical Literature database (CBM, 1978–2013.9). The word “multiple sclerosis” was entered into following websites to supplement the additional CPGs on multiple sclerosis: DynaMed (http://dynamed.ebscohost.com/), the National Guideline Clearinghouse (NGC) (http://www.guideline.gov).

### Search

A systematic and comprehensive search was performed by two reviewers. The search strategy for PubMed is presented in Appendix S1.

### Study selection

According to the inclusion and exclusion criteria, all searched records were classified using reference management software Endnote ×3 (The Thomson Reuters, Britain), and duplicate studies were discarded. Next, we read all the abstracts to identify both potentially eligible articles and any articles for which a determination could not be made from the abstract alone. Then we obtained the full-text of these articles to determine whether or not they were eligible. Study selection was independently performed by two reviewers and disagreements between reviewers were resolved through consensus or by consulting the third expert adjudicator.

### Data collection process and data items

An abstractive data extraction form was developed, piloted and modified as necessary. Two reviewers independently extracted the data and disagreements were resolved by discussion or the involvement of a third arbitrator. The extraction data included CPG characteristics (title, year of publication, organizations or countries of publication, number of authors, number of organizations, updated/period, developed methods, number of references, topics covered, number of pages) and the 23 items of AGREE II.

### Quality evaluation

A training exercise was conducted prior to commencing the quality evaluations by using a random sample of 5 CPGs. After discussion of the disagreements, two trained reviewers independently evaluated the validity of each CPG using the AGREE II instrument. The instrument consists of 23 items organized in six domains: scope and purpose, stakeholder involvement, rigor of development, clarity and presentation, applicability, and editorial independence [8]. Each item was scored from 1 (strongly disagree) to 7 (strongly agree). The score for each domain was obtained by summing all the scores of the individual items in a domain and then standardizing as follows: (obtained score - minimal possible score)/(maximal possible score - minimal possible score). The minimum standardized score for each domain was 0% and the maximum was 100%. A guideline is “strongly recommended” if the majority of items (above 4 items) scored above 50%. A guideline is “recommended” if 3 main items scored above 50%. A guideline is “not recommended” if all items scored below 50%.

### Synthesis of results

A descriptive statistical analysis for each domain was performed. Descriptive values include percentage, mean, and standard deviation (SD). Inter-rater reliability within each domain was determined by the Intraclass Correlation Coefficients (ICCs) with a 95% CI. The degree of agreement was classified according to the following scale proposed by Landis and Koch: poor (<0.00), slight (between 0.00 and 0.20), fair (from 0.21 to 0.40), moderate (from 0.41 to 0.60), substantial (from 0.61 to 0.80) and very good or almost perfect (from 0.81 to 1.00) [9]. Statistical significance was set at P<0.05. The software used for analysis was SPSS 17.0.

In addition, the overall domain scores were compared according to type of CPG, date of publication, performers, country/region, number of authors, updates, topics covered and whether it is a guideline or consensus.

## Results

### Study selection

**Figure 1:**
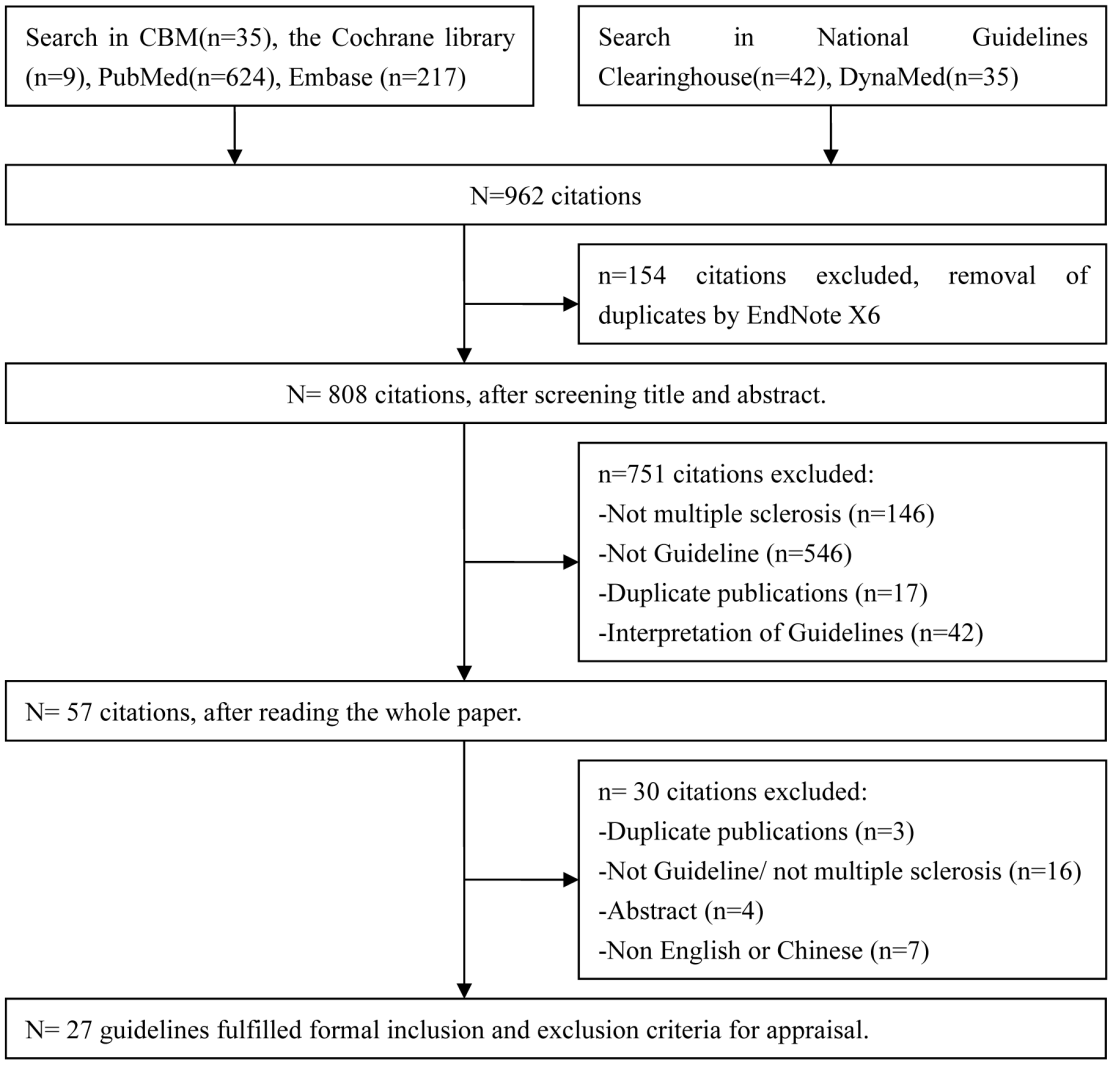
Searching and selecting guidelines flowchart.

A total of 885 citations were identified through a comprehensive database search and 77 records were searched on website related to the CPG. 905 were excluded based on the eligibility criteria previously outlined, 57 were considered for full-text screening and 27 were included in the review (Figure 1) (Appendix S2).

### Clinical practice guideline characteristics

27 CPGs were developed between 1994 and 2013, including 11 consensuses and 16 guidelines. 9 were developed by American organizations, and 11 were developed by European organizations. 12 of CPGs (44.44%) were evidence-based guidelines. The majority of CPGs (96.30%) reported the number of authors, 14 of the CPGs (51.85%) had more than 10 authors, and the average total number of authors of a CPG was 15.42 (range: 1–86). Three of CPGs (11.11%) mentioned the time of updates. The average total number of pages of a CPG was 8.85 (range: 2–22). Twenty-five (92.59%) of CPGs cited references (mean: 72.76; range: 6–268) (Table 1).

**Table 1:** The basic characteristics for included 27 CPGs.

Category		n	%
**Year of publication**	≤2009	12	44.44
	≥2010	15	55.56
**Country/Region**	America	9	33.33
	Europe	11	40.74
	South Africa	2	7.41
	Southwest Asia	1	3.70
	China	2	7.41
	Latin America	1	3.70
	Middle East	1	3.70
**Level of development**	Professional organization	18	66.67
	Regional independent body	9	33.33
**Numbers of authors**	<10	12	44.44
	≥10	14	51.85
	Not reported	1	3.70
	Average (Range)	15.42 (1–86)	
**Updated**	Mentioned	3	11.11
	Not mentioned	24	88.89
**Topics covered**	Diagnosis	4	14.81
	Treatment	13	48.15
	Management	1	3.70
	Diagnosis + Management	4	14.81
	Diagnosis + Treatment	5	18.52
**Developed methods**	Literature review	10	37.04
	Expert consensus	4	14.81
	Evidence-based	11	40.74
	Unclear	2	7.41
**Numbers of pages**	Average (Range)	8.85 (2–22)	
**Numbers of references**	Average (Range)	72.76 (6–268)	

### AGREE II evaluation results

A total of 27 CPGs were evaluated using the AGREE II instrument, with two trained reviewers. The overall agreement between reviewers was very good for most of the AGREE II domains, including scope and purpose (ICC  = 0.846; 95%CI: 0.667–0.929), stakeholder involvement (ICC  = 0.852; 95%CI: 0.680–0.932), rigor of development (ICC  = 0.865; 95%CI: 0.708–0.938) and applicability (ICC  = 0.873; 95%CI: 0.725–0.941). The agreement between reviewers for clarity of presentation (ICC  = 0.751; 95%CI: 0.461–0.885) and editorial independence (ICC  = 0.788; 95%CI: 0.542–0.902) was substantial.

6 CPGs [10]–[15] were strongly recommended as the majority of the items (above 4 items) scored above 50%, and 5 of 6 CPGs were produced by the National Guideline Clearinghouse (NGC). One guideline [16] was recommended due to 3 main items scoring above 50%. Four CPGs [17]–[20] were not recommended because all items scored below 50%.

Overall, the CPGs received the lowest scores for applicability across all six AGREE II domains (mean score: 27.08%±17.66%, range: 4.17%–66.67%), whereas they scored highest on clarity of presentation (mean score: 60.39%±13.73%, range: 33.33%–83.33%). Table 2 compared the domain scores according to year of publication, country/region, level of development, number of authors, updates, topics covered, type of CPG, and whether it was a guideline or consensus. All domain scores of CPGs published after AGREE II instrument development and validation (2010) appeared slightly higher except for editorial independence (Domain 6). The scores were highest in clarity of presentation (Domain 4, 64.26±10.54) and lowest in editorial independence (Domain 6, 28.06±24.27) in or after 2010. Twenty-seven CPGs were from seven countries or regions. America-based and American Academy of Neurology (AAN) CPGs scored the highest for scope and purpose (Domain 1, 67.59±18.94 and 73.61±20.99) and CPGs developed by six other countries or regions scored the highest for clarity of presentation (Domain 4, 56.82±15.88 for Europe, 61.11±0.00 for South Africa, 56.94±9.82 for China, 57.41±8.49 for Southwest Asia, Latin America, and Middle East). CPGs developed by regional independent bodies received the highest scores for clarity of presentation (Domain 4, 54.94±11.18) and the lowest scores for applicability (Domain 5, 20.60±7.47). Updated CPGs received higher scores than ones that were not updated. Three updated CPGs were strongly recommended because the mean scores for all six domains were above 50%, and they scored highest on scope and purpose (Domain 1, 87.96±4.24), the lowest scores for applicability (Domain 5, 53.47±4.34). The topics for the 27 CPGs that were included covered diagnosis, treatment, and management. The stratified results showed that CPGs related to treatment received higher scores for all domains. Of the 27 CPGs assessed, 12 were evidence-based (EB) CPGs. The other 15 were considered non-EB CPGs. Table 2 shows that EB CPGs have higher quality scores for all of the AGREE domains. However, 4 of 6 domains scored below 50%, and the lowest scores appeared in applicability (Domain 5, 38.37±20.21).

**Table 2:** Domain scores (%) of included 27 CPGs according to different stratified factors.

Category		Scope & Purpose	Stakeholders	Rigor	Clarity	Applicability	Editorial
**Year of publication**	≤2009	55.32±17.06	26.85±17.62	25.43±21.17	55.56±16.07	23.78±17.14	29.51±19.90
	≥2010	62.04±15.27	31.67±18.03	36.39±21.19	64.26±10.54	29.72±18.20	28.06±24.27
**Country/Region**	America	67.59±18.94	39.20±22.54	44.56±25.63	66.36±14.26	37.27±22.06	37.96±20.98
	Europe	56.06±16.19	27.27±15.31	26.70±20.62	56.82±15.88	24.05±15.54	32.20±20.67
	South Africa	54.17±1.96	18.06±5.89	20.83±1.47	61.11±0.00	23.96±10.31	0.00±0.00
	China	48.61±5.89	26.39±9.82	20.31±3.68	56.94±9.82	13.54±1.47	0.00±0.00
	Other countries	54.63±8.93	18.52±4.24	24.65±6.70	57.41±8.49	18.75±5.51	26.39±17.35
**Level of development**	American Academy of Neurology	73.61±20.99	44.91±25.42	54.34±26.06	70.83±14.25	43.75±24.44	40.97±21.15
	Other professional organizations	55.56±14.26	27.08±15.22	28.30±17.74	59.26±13.31	23.61±15.03	24.65±22.85
	Regional independent body	54.01±9.22	22.53±7.14	20.60±10.23	54.94±11.18	20.60±7.47	25.93±20.81
**Numbers of authors**	<10	52.78±12.76	20.60±11.87	23.00±16.27	55.56±10.86	20.49±12.24	27.08±21.94
	≥10	64.88±17.52	36.90±19.29	39.43±23.68	64.29±15.44	33.63±19.96	32.14±22.01
**Updated**	Mentioned	87.96±4.24	62.04±5.78	72.22±3.94	80.56±2.78	53.47±4.34	54.17±14.43
	Not mentioned	55.44±13.04	25.46±13.97	26.43±16.68	57.87±12.36	23.78±15.77	25.52±20.86
**Topics covered**	Diagnosis	55.56±24.00	34.03±24.47	27.34±31.57	49.31±21.32	26.56±18.19	38.54±24.15
	Treatment	60.90±17.77	30.98±18.63	33.49±21.92	62.39±13.40	28.37±17.91	32.05±17.54
	Diagnosis + Management + Treatment	58.06±11.30	25.83±14.58	30.63±18.67	62.22±9.46	25.63±18.92	20.42±25.72
**Evidence-based CPGs**	Yes	68.52±18.21	40.28±20.22	46.79±22.82	69.21±11.39	38.37±20.21	39.24±22.58
	No	51.48±9.20	20.93±8.96	19.31±9.48	53.33±11.32	18.06±7.86	20.28±18.15
**Guideline/Consensus**	Guideline	64.41±17.89	34.03±21.00	39.00±24.39	66.32±13.60	33.33±19.65	34.11±25.19
	Consensus	51.26±8.90	22.98±8.43	20.64±9.50	51.77±8.63	17.99±8.85	20.83±13.94
**Overall scores**		59.05±16.13	29.53±17.67	31.52±21.50	60.39±13.73	27.08±17.66	28.70±22.03

## Discussion

This is the first study to systematically evaluate the methodological quality of CPGs on diagnosis, treatment, and management of MS published in English and Chinese. For the most part, the quality scores for scope and purpose (59.05%) and clarity of presentation (60.39%) are acceptable. However, the methodological quality of the CPGs in the study had some flaws, including the representation of all stakeholders (consumers, all relevant professional group, target users, 29.53%), developing guidelines with scientific rigor (31.52%), supporting implementation of the recommendations (27.08%), and declaring editorial independence (28.70%). Our results are similar to the study conducted by Alonso-Coello P et al.'s which assessed a total of 626 CPGs on different topics and showed that the mean quality scores were moderate (43% for rigor of development) to low (35% for stakeholder involvement, 30% for editorial independence, and 20% for applicability) [21]. 22.22% of the CPGs were recommended strongly because the majority of the items (above 4 items) scored above 50%, and 14.81% of CPGs were not recommended because all of the items scored below 50%. The results of a stratified analysis show that all domain scores of CPGs published in or after 2010 appear slightly higher except for editorial independence. The mean scores of all six domains are higher for CPGs developed by American organizations and AAN, CPGs with more than ten authors, updated CPGs, EB CPGs, and guidelines rather than consensuses.

There were serious methodological reporting flaws for the included CPGs in the items of stakeholder involvement, rigor of development, applicability and editorial independence. Most of CPGs lacked explicit statements on the views and preferences of the target population (e.g., patients, public, etc.) (item 5), but the target users of guidelines were well-defined (item 6). Rigor of development is considered to be the most important domain and more attention should be made to whether external reviews are performed before CPGs are published (item 13) and whether updating mechanisms for the guidelines are provided (item 14). However, the quality of the “applicability” domain also plays a critical role in the implementation of a guideline. An effective guideline should provide advice as to how the recommendations can be implemented present discussion on the potential impact of recommendations on resources and requires clearly defined criteria derived from the key recommendations [8]. Unfortunately, flaws in CPGs were found in two items including whether or not the guidelines describe facilitators and barriers to their application (item 18) and whether or not the potential resource implications of applying the recommendations have been considered (item 20). The AGREE II instrument is used for the rigor and transparency of CPG development and to suggest how to improve existing CPGs [8], and it requires developers of guidelines to report potential conflicts of interest. Our results show that there are serious reporting flaws for potential conflicts of interest for the members of the guideline development group (item 23).

Our study has several strengths. First, the latest instrument for guidelines assessment (AGREE II) was used to assess the methodological quality of CPGs related to MS. Second, we performed a stratified analysis and found the potential elements that most significantly influenced CPG quality. Third, we conducted a systematic and comprehensive literature searching, including three main English academic databases (PubMed, EMBASE, Cochrane Library), two web-based searches related to CPGs (NGC, and DynaMed), and one Chinese database (CBM). Lastly, the inter-reviewer consensus was high (above 70%), so our conclusions are reliable.

On the other hand, some limitations are noted in our study. First, although the processes of searching, study selection, data extraction and quality assessment were conducted independently by two reviewers, there are still some limitations due to the different level of understanding of the AGREE II instrument the two reviewers have. Second, we only included CPGs in English and Chinese, so CPGsin other languages were not considered. Third, this review only assessed the reporting of the different items and not the content validity of the recommendations. Finally, other instruments such as the four-item Global Rating Scale (GRS), which plays an important role in guideline evaluation, should be considered [22]. Although the GRS is less sensitive than the AGREE-II in detecting differences in guideline quality, its items did predict outcome measures related to guideline adoption [23].

Overall, the quality of CPGs on MS was acceptable for scope and purpose and clarity of presentation. The developers of CPGs need to pay more attention to editorial independence, applicability, rigor of development, and stakeholder involvement during the development process. The AGREE II instrument should be adopted by guideline developers.

## Supporting Information

Appendix S1 **Search algorithms for PubMed.**
(DOC)

Appendix S2 **Included CPGs(n = 27).**
(DOC)

Checklist S1 **PRISMA Checklist.**
(DOC)
