# Supplementary material for: Systematic Review of Clinical Practice Guidelines Related to Multiple Sclerosis
Source: PLoS One. 2014 Oct 10;9(10):e106762. doi: 10.1371/journal.pone.0106762 (PMC4193735; doi:10.1371/journal.pone.0106762)
Supplement: Appendix S1 — Search algorithms for PubMed. (DOC) [file pone.0106762.s001.doc]

**Search strategy for PubMed:**

#1 ‘‘multiple sclerosis’’[MeSH Terms]

#2 “multiple sclerosis’’[Title]

#3 "disseminated sclerosis"[Title]

#4 #1 OR #2 OR #3

#5 Consensus Development Conference[ptyp]

#6 Consensus Development Conference, NIH[ptyp]

#7 Guideline[ptyp]

#8 Practice Guideline[ptyp]

#9 guideline*[Title/Abstract]

#10 consensus[Title/Abstract]

#11 #5 OR #6 OR #7 OR #8 OR #9 OR #10

#12 #4 AND #11
