# Supplementary material for: Systematic Review of Clinical Practice Guidelines Related to Multiple Sclerosis
Source: PLoS One. 2014 Oct 10;9(10):e106762. doi: 10.1371/journal.pone.0106762 (PMC4193735; doi:10.1371/journal.pone.0106762)
Supplement: Appendix S2 — Included CPGs(n = 27). (DOC) [file pone.0106762.s002.doc]

Appendix2 Included CPGs(n=27)

| **Title** | **Year** |
| --- | --- |
| Recommended Diagnostic Criteria for Multiple Sclerosis: Guidelines from the International Panel on the Diagnosis of Multiple Sclerosis | 2001 |
| Expert opinion: Guidelines for the use of natalizumab in multiple sclerosis patients previously treated with immunomodulating therapies | 2007 |
| Intense immunosuppression in patients with rapidly worsening multiple sclerosis: treatment guidelines for the clinician | 2008 |
| Consensus statement on medication use in multiple sclerosis bythe Spanish Society of Neurology’s study group for demyelinating diseases | 2013 |
| Recommendations for diagnosis and management of multiple sclerosis | 2012 |
| Consensus statement: evaluation of new and existing therapeutics for pediatric multiple sclerosis | 2011 |
| Guideline for the diagnosis and management of multiple sclerosis: A Southern African perspective | 2013 |
| Guideline for the diagnosis and management of multiple sclerosis in children | 2013 |
| Consensus guidelines for the diagnosis and treatment of multiple sclerosis | 2013 |
| Guidelines for autologous blood and marrow stem cell transplantation in multiple sclerosis: a consensus report written on behalf of the European Group for Blood and Marrow Transplantation and the European Charcot Foundation | 2000 |
| Guidelines for using proton MR spectroscopy in multicenter clinical MS studies | 2007 |
| New therapies in multiple sclerosis: beta-interferon | 1996 |
| Cerebrospinal fluid in the diagnosis of multiple sclerosis: a consensus report | 1994 |
| EFNS guidelines on diagnosis and management of neuromyelitis optica | 2010 |
| The therapeutic potential of mesenchymal stem cell transplantation as a treatment for multiple sclerosis: consensus report of the International MSCT Study Group | 2010 |
| Assessment: The use of natalizumab (Tysabri) for the treatment of multiple sclerosis (an evidence-based review): Report of the Therapeutics and Technology Assessment Subcommittee of the American Academy of Neurology | 2008 |
| Disease modifying therapies in multiple sclerosis: report of the therapeutics and technology assessment subcommittee of the american academy of neurology and the MS council for clinical practice guidelines | 2002 |
| Assessment: The use of natalizumab (Tysabri) for the treatment of multiple sclerosis (an evidence-based review): Report of the Therapeutics and Technology Assessment Subcommittee of the American Academy of Neurology | 2008 |
| Evidence report: the efficacy and safety of mitoxantrone (Novantrone) in the treatment of multiple sclerosis | 2010 |
| Use of imaging in multiple sclerosis | 2011 |
| Nursing management of the patient with multiple sclerosis | 2011 |
| Optimizing outcomes in multiple sclerosis: consensus guidelines for the diagnosis and treatment of multiple sclerosis in Latin America | 2011 |
| Basic and escalating immunomodulatory treatments in multiple sclerosis: Current therapeutic recommendations | 2008 |
| Consensus of diagnosis and treatment for Multiple Sclerosis | 2012 |
| Expert consensus of diagnosis and treatment for Multiple Sclerosis in China | 2010 |
| Guidelines for differential diagnosis of suspected multiple sclerosis | 2009 |
| Adherence to Disease-Modifying Drugs in Patients with Multiple Sclerosis: A Consensus Statement from the Middle East MS Advisory Group | 2010 |
